# Abdomino-Vaginal Impalement

**Published:** 1870-12

**Authors:** 


Abdomino-Vaginal Impalement.—Dr. W. M. James tells of a case of accident of this character that is both curious and instructive.

A woman weighing nearly 200 pounds, climbs a hay-mow in search of eggs; leaping down on a pile of hay, there chances to be a pitchfork projecting, handle upward, upon which she falls. The handle of the fork enters the vagina, plunges through the vaginal wall, making a rupture just at the right of the mouth of the womb and behind it; it passes upward through the abdominal cavity until it strikes the ninth or tenth rib, near the spinal column on the left side. The handle was arrested by the ribs, and held so firmly that the woman was carried over at right angles from the mow, the whole weight bearing on the instrument.

After all this violence within the abdominal cavity, the woman made steady improvement and went on to complete recovery. Opium was freely used, and disinfectants to the vagina.

After the vaginal wound healed, an abscess formed in the region of the injured rib, which was opened externally.—*Journal Gynæcological Society.*

